# Epilepsy and Its Interaction With Sleep and Circadian Rhythm

**DOI:** 10.3389/fneur.2020.00327

**Published:** 2020-05-08

**Authors:** Bo Jin, Thandar Aung, Yu Geng, Shuang Wang

**Affiliations:** ^1^Department of Neurology, Zhejiang Provincial People's Hospital, People's Hospital of Hangzhou Medical College, Hangzhou, China; ^2^Barrow Neurological Institute, Epilepsy Center, Phoenix, AZ, United States; ^3^Department of Neurology, Epilepsy Center, Second Affiliated Hospital, School of Medicine, Zhejiang University, Hangzhou, China

**Keywords:** epilepsy, sleep, circadian rhythms, chronotherapy, seizure forecasting

## Abstract

Growing evidence shows the bidirectional interactions between sleep, circadian rhythm, and epilepsy. Comprehending how these interact with each other may help to advance our understanding of the pathophysiology of epilepsy and develop new treatment strategies to improve seizure control by reducing the medication side effects and the risks associated with seizures. In this review, we present the overview of different temporal patterns of interictal epileptiform discharges and epileptic seizures over a period of 24 consecutive hours. Furthermore, we discuss the underlying mechanism of the core-clock gene in periodic seizure occurrences. Finally, we outline the role of circadian patterns of seizures on seizure forecasting models and its implication for chronotherapy in epilepsy.

Bidirectional interactions between sleep and epilepsy have been recognized for centuries. Patients with epilepsy often have associated co-morbid major sleep disorders such as obstructive sleep apnea (1). On the other hand, sleep disruption is one of the major risk factors for the likelihood of recurrent seizures (2). In addition, the circadian rhythm itself seems to influence the timing and the severity of epileptic seizures (3). Unraveling the underlying mechanism of these interactions may lead to new treatment strategies for epilepsy. Here we comprehensively review the recent advances in the relationship between circadian rhythms, sleep, and epilepsy.

## A Brief Introduction to Circadian Rhythm

The circadian rhythm refers to the 24-h cycle of behavioral and physiological activity. The circadian rhythmicity of mammals is a hierarchically organized multi-oscillator system, coordinated by a central pacemaker located in the suprachiasmatic nucleus of the hypothalamus (4, 5). The molecular mechanism underlying circadian rhythm are cell-autonomous transcription–translation feedback loops comprising a set of core-clock genes: brain muscle aryl nuclear translocase like-1 (BMAL1), circadian locomotor output cycles kaput (CLOCK), Period (PER1, PER2, and PER3), and cryptochrome (CRY1 and CRY2) (6). The BMAL1 and CLOCK heterodimerize during the early circadian day and activate the transcription of PER and CRY with promoters containing circadian E-box elements. During the early circadian night, complexes containing PER and CRY repress BMAL1–CLOCK-mediated transcription. During the later circadian night, the PER–CRY complexes degrade, and the cycle starts again (5, 7). In addition, BMAL1–CLOCK complexes activate the expression of nuclear receptors REV-ERBα and ROR, which ultimately compete to repress or activate BMAL1 transcription, respectively (8, 9). Complementary to these feedback loops, the circadian rhythm is modulated by various regulations at the post-transcriptional, translational, and post-translational levels to fine-tune the circadian system (10–12). The details of their exact role and function are beyond the scope of this review, but they can be found in previously published articles (13).

## Core-Clock Gene and Epilepsy

The core-clock genes not only regulate the circadian rhythms (14, 15) but also contribute to epileptic susceptibility. The core-clock genes can disrupt the neuronal inhibitory and the excitatory balance rhythmically, leading to seizure periodicity (16–18). In a post-status epilepticus animal model of mesial temporal lobe epilepsy, the expression of core-clock genes is found to be dysregulated during epileptogenesis: the expression of PER1 and CRY1 increases in the early post-status epilepticus condition compared to the control condition and decreases to the baseline level in epileptic condition; the expression of BMAL1, CLOCK, and CRY2 gradually decreases during epileptogenesis (17). However, the exact role of the core-clock genes in the process of epileptogenesis remains unknown. Future studies with functional assays may give us more insight. Besides that, in the animal model of generalized seizure induced by electrical stimulation, the circadian profile of seizure threshold is absent in BMAL1 knock-out mice and its seizure threshold is significantly lower compared to that of the wild-type mice (19). Interestingly, the expression of the CLOCK gene in epileptic brain specimens, obtained from focal cortical dysplasia (FCD), and tuberous sclerosis complex (TSC) cases, is found to be reduced in epileptogenic tissue when compared to control tissue. In addition, Emx- Cre, Clockflox/flox mice with conditional deletion of CLOCK in the excitatory neurons, exhibits sleep-related seizures (20). Those results suggest that the disruption of the function of the core-clock genes may play an important role in the generation of focal epilepsy. However, most experiments are conducted on temporal lobe epilepsy; further studies on extratemporal lobe epilepsy are warranted.

The mammalian target of rapamycin (mTOR) signaling pathway is a critical regulator of protein and lipid synthesis, growth, and proliferation of cells (21). A series of recent publications have shown that the mTOR pathway is overactivated in the epileptic tissue obtained from temporal lobe epilepsy with hippocampal sclerosis and FCD (22, 23). Interestingly, several results have shown that there is a close interaction between core-clock genes and the mTOR signaling pathway. Circadian rhythmicity can be observed in the mTOR signaling pathway (24, 25). Furthermore, BMAL1, which is activated by the mTOR complex 1 kinase pathway via phosphorylation, regulates protein synthesis (26). The circadian rhythm abnormalities of TSC mouse and cellular models are controlled by increased translation and decreased degradation of BMAL1 (27). Additionally, BMAL1 negatively regulates mTOR complex 1, and this regulation is essential for aging (25). Taking these findings together, we speculate that core-clock genes might play an essential role in the type of epilepsy associated with mTORopathies. Thus, core-clock genes may be considered as the potential innovative targets for epilepsy treatment.

## Modulation of Interictal Epileptiform Discharges and Seizures by Sleep and Circadian Rhythm

At the end of the 19th century, Gowers reported that 21% of patient's seizures were nocturnal types, 42% were diurnal types, and 37% were mixed types (28). The wide usage of long-term video electroencephalography (EEG) monitoring dramatically improves our knowledge about the role of circadian rhythmicity in epilepsy. It has been reported that interictal epileptiform discharges (IEDs) and epileptic seizures tend to occur in a specific temporal pattern according to the type as well as the localization of epilepsy (29, 30). A meta-analysis based on 10 publications using conventional and intracranial EEG revealed that, compared to rapid eye movement (REM) sleep, the relative focal IED occurrence rate was 1.11 times higher in wakefulness, 1.75 times higher in stage one of non-REM (NREM) sleep, 1.69 times higher in stage two of NREM sleep, and 2.46 times higher in stage three of NREM sleep (31). These data suggest that IEDs are activated during NREM sleep. Moreover, it has been well-known that individual IEDs become more extensive and widespread during NREM sleep stages. Quite the reverse, IEDs are relatively suppressed and restricted during the REM sleep stage, which can be more accurate in localizing the epileptogenic zone (32–34). The above findings can be explained by the fact that increased EEG synchronization is observed during NREM sleep due to slow oscillations, and the increased EEG desynchronization is noted during REM sleep due to cholinergic modulation (35, 36). Additionally, there is increasing evidence that the distribution of IEDs is not homogenous during both NREM sleep and REM sleep. The occurrence of IEDs during NREM sleep is associated with the cyclic alternating pattern, which is characterized by the regular alternation of the two EEG patterns: arousal complex phase A and post-arousal rebound response phase B. Phase A usually lasts 8–15 s and is composed of EEG rhythms of sleep oscillating with periodic stage-related arousal complexes such as K complexes, bursts of delta waves, and arousals. Phase A is followed by the interval of 15–20-s-long inhibitory phase B, which is composed of stage-related background activity (37). Furthermore, IEDs occur more frequently in phase A (38). In addition, Frasuscher reported that IEDs preferentially occurred during high-amplitude widespread slow waves of NREM, especially during the transition from the “up” (activated state, when the pyramidal neurons were depolarized) to the “down” (deactivated state, when the pyramidal neurons were hyperpolarized) state of the slow wave (39). Later, Ujma and colleagues validated that IEDs occurred more frequently during the highly synchronous NREM sleep periods, denoted by steeper slopes and higher-amplitude slow waves (40). Thus, EEG synchronization is the underlying factor for enhancing IED activity during NREM sleep. In regards to REM sleep, phasic REM sleep (increased EEG desynchronization mediated by high cholinergic neurotransmission) has a more enhanced suppression effect on IEDs than tonic REM sleep (decreased EEG desynchronization mediated by low cholinergic neurotransmission), highlighting the role of EEG desynchronization in the suppression of IEDs (34, 36).

Additionally, the latest clinical application of closed-loop implantable brain stimulator offers a unique opportunity to monitor human brain activity. Spencer and colleagues analyzed the intracranial EEG data of 134 epileptic patients implanted with the NeuroPace RNS system over an 84-day period. They reported that IEDs had a robust non-random circadian pattern, which uniformly peaked during regular sleep hours regardless of the location of the seizure-onset zone (41). Another study reported that IEDs had a multiday period (commonly 20–30 days in duration), and the period was relatively stable for up to 10 years in 37 epileptic patients implanted with similar devices (3).

Depending on the localization of the seizure-onset zone, seizures occur at different circadian patterns. Seizures that originated in the frontal lobe occur predominantly during sleep, while temporal and occipital seizures are prevalent during wakefulness. Parietal seizures, on the other hand, vary across different studies (29, 42). Furthermore, the circadian rhythm of seizure onset is also influenced by seizure types. Tonic, tonic-clonic, and hypermotor seizures occur more frequently during sleep, whereas, clonic, myoclonic, and hypomotor seizures are more often reported to occur during daytime (29). Additionally, clusters of isolated epileptic seizures occur more predominantly during daytime compared with isolated seizures (43). A study utilizing two comprehensive databases of human seizures (SeizureTracker and NeuroVista) further confirmed that the seizure activity was modulated by different rhythmic cycles such as circadian rhythms, which were the most common pattern, followed by weekly rhythms and monthly rhythms (30). Similar results were replicated in a different study conducted by Baud et al. (3). Interestingly, men and women have a similar pattern of seizure cycling, suggesting that those rhythms are driven by some mechanisms other than menstruation (3, 30).

Evidence from the epileptic animal model also shows similar results. A pilocarpine-induced mesial temporal lobe epileptic mouse model shows that seizure clusters occur in a circadian pattern between 4 and 7 p.m. (44). The chronic EEG data, recorded over 30 days in kainite- and pilocarpine-induced epilepsy models, reveal that not only circadian rhythms but also multi-day rhythms modulate the occurrence of IEDs and epileptic seizures (5–7 days and 6 days, respectively). Moreover, those rhythms are not influenced by external intervention, which suggested that endogenous factors rather than environmental effects regulate this phenomenon (45). It is clear that both sleep–wake patterns and circadian rhythms or multi-day rhythms play an important role in the generation of IEDs and seizure; thus, epilepsy may be considered as a cyclical disorder (46).

## Sleep-Related Epilepsy

The spectrum of the idiopathic focal children epilepsies ranges from the most benign type, benign epilepsy with centrotemporal spikes (BECTs), to the extreme malignant category, Landau–Kleffner syndrome (LKS) and electrical status epilepticus during slow-wave sleep (ESES) (47, 48), which are known to have a close relationship with sleep. BECT is the most common focal epileptic syndrome in children and may associate with mild cognitive dysfunctions (49). The seizures usually occur during sleep as sensorimotor manifestation of face, tongue, and/or throat, less frequently, followed by secondary generalization with frequent unilateral or bilateral synchronous centrotemporal discharges on the interictal EEG (50). During NREM sleep, the centrotemporal discharges become not only higher in amplitude but also more frequent (48). Accompanied by the increased frequency of seizures and IEDs, sleep architecture is affected, along with a decrease in the proportion of REM sleep and a longer REM sleep-onset latency (51).

Patients with ESES have global or selective developmental regression, motor impairment, and multiple seizure types, with EEG showing nearly continuous (>85%) interictal epileptiform discharges (either focal, bilateral independent, or bilateral synchronous) throughout NREM sleep regardless of etiology (52). Patients with BECTs can progress into a malignant epileptic encephalopathy in the form of ESES (53, 54). LKS is the subset of ESES patients with pronounced language regression as in acquired childhood epileptic aphasia (52). All of them involve the perisylvian network called the “perisylvian neuronal network epilepsies” (55, 56). One of the most critical features in this epilepsy spectrum is the propensity to increase IEDs during NREM sleep, resulting in cognitive impairment (55, 57). IEDs during NREM sleep interfere with sleep plastic functions, compromising the “refreshment” of synapses that may explain the decline of cognitive function (57–60).

Juvenile myoclonic epilepsy (JME) is one of the most common types of idiopathic generalized epilepsy. The myoclonic jerks usually occur on in the morning shortly after awaking, and generalized tonic-clonic seizures can occur independently or precede the myoclonus (61). The patients may be exceedingly sensitive to sleep deprivation. With transcranial magnetic stimulation, patients with JME have prominent increases in cortical excitability compared with healthy controls or patients with focal epilepsy, especially during the early morning (62). It may be the explanation for increased seizure susceptibility in JME patients at this time of the day. In addition, the REM sleep-onset latency is prolonged, and the REM percentage is decreased (63). Thus, there are significant alterations in sleep architecture in patients with JME.

There is a close relationship between sleep and FCD type II (64, 65). The pathological substrate of FCD type II increases the risk of sleep-related epilepsy with respect to other histopathological substrates, regardless of its location (64). Interestingly, a small FCD type II lesion is associated with a higher proportion of sleep-related epilepsy compared with a larger FCD type II lesion (66). The lesion of FCD type II is usually confined, which offers a unique opportunity to explore the interrelationship between sleep and epileptic activity. As revealed by intracranial EEG recordings, the influence of the sleep–wake process on FCD type II is evident. During wakefulness, sub-continuous spikes and polyspikes, which is the most common intracranial interictal activity produced by intralesional FCD type II, indicate a state of reduced possibility for seizure generation. These patterns are replaced by frequent short bursts of low-voltage fast discharges during NREM sleep, which is suggestive of a reduced modulation of the FCD type II by the surrounding cortex, and therefore it might play a pivotal role in seizure initiation (67, 68). As the CLOCK gene and the mTOR signaling pathways are two essential regulatory systems in the modulation of seizure thresholds in patients with FCD type II, further studies are required to explore the interaction between CLOCK gene and mTOR signaling pathways in order to unravel the pathogenetic mechanisms of FCD type II.

## Seizure Forecasting

Seizure prediction can prevent or reduce the risk of a seizure and its complication, and it has been explored for decades. Karoly et al. analyzed nine patients' chronic intracranial EEG data and illustrated that the incorporation of the individual circadian patterns of seizures into EEG signals could greatly improve the accuracy of the seizure prediction algorithms (69). Baud et al. reported that seizures occurred preferentially during the rising phase of multi-day IED rhythms (3). Thus, adding patient-specific multi-day IED rhythms may further enhance the accuracy of seizure prediction algorithms. However, only a small portion of patients with epilepsy have these intracranial devices, and it may be impossible to generalize these aspects of seizures to all patients with epilepsy. Further investigations are crucial to find a better way to predict seizure occurrence using non-invasive devices, including the factors which have a close relationship to the timing of seizure occurrence such as seizure-onset zone and seizure types.

## Treatment Strategies

Despite the advent of several new antiepileptic drugs (AEDs), almost 30–40% of patients with epilepsy continue to have seizures (70). However, among pharmaco-resistant epilepsy, only a minority of patients qualify for resective epilepsy surgery. There is an urgent medical necessity to provide better seizure control in those patients. Chronotherapy is a therapeutic intervention to enhance the effectiveness and the tolerance of a drug by determining optimal medication administration times from a circadian perspective (71). Multiple studies show promising results in optimizing seizure frequency in patients with epilepsy. By discerning the temporal pattern of seizure occurrence, chronotherapy can be accomplished by administrating the conventional drug at appropriate timing. Besides that, as there is a close relationship between clock genes and epilepsy, chronotherapy can also be achieved by the application of external cues that reset the circadian rhythms.

One study reported that the efficacy of different timings of carbamazepine and phenytoin intake had different influences on seizure control in 103 epileptic patients. In the study, the patients who took the majority of AEDs (two-thirds or three-fourths) at 8 pm. achieved better seizure control than the patients who were treated with twice-daily regimens. Also, the former group showed better drug tolerance than the latter group (72). Another study revealed that an improvement in seizure control was noted in patients with persistent nocturnal or early-morning seizures after taking a higher AED dose at bedtime, and only 11.8% of the patients experienced fatigue (73). Future studies are necessary for patients who have multi-day rhythms to investigate the effectiveness and the tolerance of the unique treatment by titrating the patients' own AED peak dose according to the higher seizure risk days and the lower seizure risk days.

Melatonin is used as a chronotherapeutic medication as it modulates circadian rhythm. Several studies have investigated the role of melatonin in patients with epilepsy. However, the findings are inconsistent across the different studies, and no specific conclusion can be drawn about the role of melatonin in seizure control (74). Moreover, light can reset the clock system even by quick flash exposures (75). A double-blinded randomized control trial demonstrated that light therapy could reduce the seizure frequency in patients with drug-intractable temporal lobe epilepsy. However, in the same study, the author described that light therapy could exaggerate seizure activity in patients with extratemporal lobe epilepsy (76). Chronotherapy appears to offer promising results in epilepsy management, but data on chronotherapy are still scarce. Further studies, including randomized controlled trials, are needed in the role of chronotherapy in epilepsy management.

Chronopharmacokinetics is the study of changes in the absorption, metabolism, distribution, and elimination of drugs in the body (77). The drug pharmacokinetic properties can be affected by many factors, such as dietary and circadian clock genes (78). A well-known example of chronopharmacokinetic is that the absorption of valproate can be delayed for hours if the medication is taken after a meal (79). Another example is the diurnal expression of the CYP3A4 gene that is required for the metabolism of carbamazepine (80). In turn, AEDs may have profound effects on circadian rhythms. Griggs et al. demonstrated that valproic acid could disrupt the oscillatory expression of circadian rhythm transcription factors in cell cultures (81). However, whether or to what extent the chronobiological effects of AEDs contribute to seizure frequency and severity remains unknown, and future studies are needed to explore this exciting topic.

## Conclusions

Long-term EEG video monitoring and the clinical application of closed-loop implantable brain stimulators have greatly enhanced our knowledge of the interaction between circadian rhythms, sleep, and epilepsy. Clock genes such as CLOCK may play an essential role in the generation of focal epilepsy, such as FCD type II, which is paradigmatic mTOR-opathy. These disorders can be considered as human models which may help us to better understand the reciprocal relationship between the clock genes and the mTOR signaling pathway. Although chronotherapy can reduce seizure frequency and the side effects of AEDs, further clinical trials with bigger sample sizes are necessary. Before this treatment strategy can be utilized in the clinical setting, multi-center studies, or randomized controlled trials are warranted. Adding individual circadian rhythms in seizure prediction algorithms is promising. If these algorithms were readily available from the non-invasive EEG data, a significant number of patients would benefit from it.

## Author Contributions

BJ contributed to the conception and drafting of the manuscript. TA and YG revised the manuscript. SW drafted the manuscript and the final approved version to be published.

## Conflict of Interest

The authors declare that the research was conducted in the absence of any commercial or financial relationships that could be construed as a potential conflict of interest.
